# Cone beam computed tomography in implant dentistry: recommendations for clinical use

**DOI:** 10.1186/s12903-018-0523-5

**Published:** 2018-05-15

**Authors:** Reinhilde Jacobs, Benjamin Salmon, Marina Codari, Bassam Hassan, Michael M. Bornstein

**Affiliations:** 1 OMFS IMPATH Research Group, Department of Imaging and Pathology, Faculty of Medicine, University of Leuven, Kapucijnenvoer 33, 3000 Leuven, Belgium; 2 Department of Oral and Maxillofacial Surgery, University Hospitals Leuven, Leuven, Belgium; 3 Department of Dental Medicine (DENTMED), Karolinska Institutet, Stockholm, Sweden; 4 EA2496, Orofacial Pathologies, Imaging and Biotherapies Lab, Dental School Paris Descartes University, Sorbonne Paris Cité, Paris, France; 5 Department of Odontology, AP-HP, Nord Val de Seine Hospital (Bretonneau), Paris, France; 6 Unit of Radiology, IRCCS Policlinico San Donato, San Donato Milanese, Italy; 7 Department of Oral Function and Restorative Dentistry, Academic Centre for Dentistry Amsterdam (ACTA), Research Institute MOVE, 1081 LA Amsterdam, The Netherlands; 8 Applied Oral Sciences, Faculty of Dentistry, The University of Hong Kong, Hong Kong SAR, China

**Keywords:** Cone beam computed tomography, Dental implants, Presurgical planning, Guidelines, Radiation dose, Virtual patient

## Abstract

**Background:**

In implant dentistry, three-dimensional (3D) imaging can be realised by dental cone beam computed tomography (CBCT), offering volumetric data on jaw bones and teeth with relatively low radiation doses and costs. The latter may explain why the market has been steadily growing since the first dental CBCT system appeared two decades ago. More than 85 different CBCT devices are currently available and this exponential growth has created a gap between scientific evidence and existing CBCT machines. Indeed, research for one CBCT machine cannot be automatically applied to other systems.

**Methods:**

Supported by a narrative review, recommendations for justified and optimized CBCT imaging in oral implant dentistry are provided.

**Results:**

The huge range in dose and diagnostic image quality requires further optimization and justification prior to clinical use. Yet, indications in implant dentistry may go beyond diagnostics. In fact, the inherent 3D datasets may further allow surgical planning and transfer to surgery via 3D printing or navigation. Nonetheless, effective radiation doses of distinct dental CBCT machines and protocols may largely vary with equivalent doses ranging between 2 to 200 panoramic radiographs, even for similar indications. Likewise, such variation is also noticed for diagnostic image quality, which reveals a massive variability amongst CBCT technologies and exposure protocols. For anatomical model making, the so-called segmentation accuracy may reach up to 200 μm, but considering wide variations in machine performance, larger inaccuracies may apply. This also holds true for linear measures, with accuracies of 200 μm being feasible, while sometimes fivefold inaccuracy levels may be reached. Diagnostic image quality may also be dramatically hampered by patient factors, such as motion and metal artefacts. Apart from radiodiagnostic possibilities, CBCT may offer a huge therapeutic potential, related to surgical guides and further prosthetic rehabilitation. Those additional opportunities may surely clarify part of the success of using CBCT for presurgical implant planning and its transfer to surgery and prosthetic solutions.

**Conclusions:**

Hence, dental CBCT could be justified for presurgical diagnosis, preoperative planning and peroperative transfer for oral implant rehabilitation, whilst striving for optimisation of CBCT based machine-dependent, patient-specific and indication-oriented variables.

## Background

Radiography is considered the most frequent diagnostic tool in daily dental practice, with more than one quarter of all medical radiographs in Europe being made by dentists. Since the discovery of x-rays 120 years ago, dental radiographs have been the predominant source of diagnostic information in the oral and maxillofacial complex. Yet, two-dimensional (2D) imaging techniques are unable to depict complicated three-dimensional (3D) anatomical structures and related pathologies.

In the nineties, there was a growing tendency in using 3D information as an aid for dentomaxillofacial diagnosis and treatment, while in the nillies, cone beam computed tomography (CBCT) imaging started to offer a solution for this growth by being made available in specialty clinics [1], These developments went hand in hand with the increasing use of 3D imaging applications for presurgical planning and transfer of oral implant treatment [2–4]. While the required 3D acquisition for dental applications was initially realized by medical computed tomography (CT), dental CBCT rapidly took over [1, 5]. The main reasons for the triumph of CBCT are its capabilities of volumetric jaw bone imaging at reasonable costs and doses, with a relative advantage of having a compact, affordable, and nearby or in-house equipment. For the clinicians involved in implant rehabilitation, the power of a dental 3D dataset is not only situated in the diagnostic field, but also in the potential of gathering integrated patient information for presurgical and treatment applications related to oral implant placement. Nowadays, rapid advances of digital technology and computer-aided design/computer-aided manufacturing (CAD/CAM) systems are indeed creating challenging opportunities for diagnosis, surgical implant planning and delivery of implant-supported prostheses. While there is still a huge demand for maximised integration of 3D datasets acquired from various imaging sources, there is also a call for simplified solutions. Yet, when striving for optimized patient-specific implant rehabilitation, the ultimate goal remains to fully integrate the available 3D imaging data thus creating the virtual patient, aiding presurgical simulation and peroperative transfer to the surgical field with further prosthetic rehabilitation [1, 5].

The aim of the present state-of-the-art paper is to present a narrative review providing support for the hypothesis on using CBCT for oral implant planning and to attempt formulating recommendations for justified and optimized CBCT imaging. Requirements for optimized use of CBCT and the related limitations are presented including a maximized use of available 3D CBCT data.

## Methods

In order to find the relevant literature included in this article, an electronic search of MEDLINE (PubMed) database was performed. This literature search included studies published in English language or with an English language abstract published prior to November 30th, 2016.

**Fig. 1:**
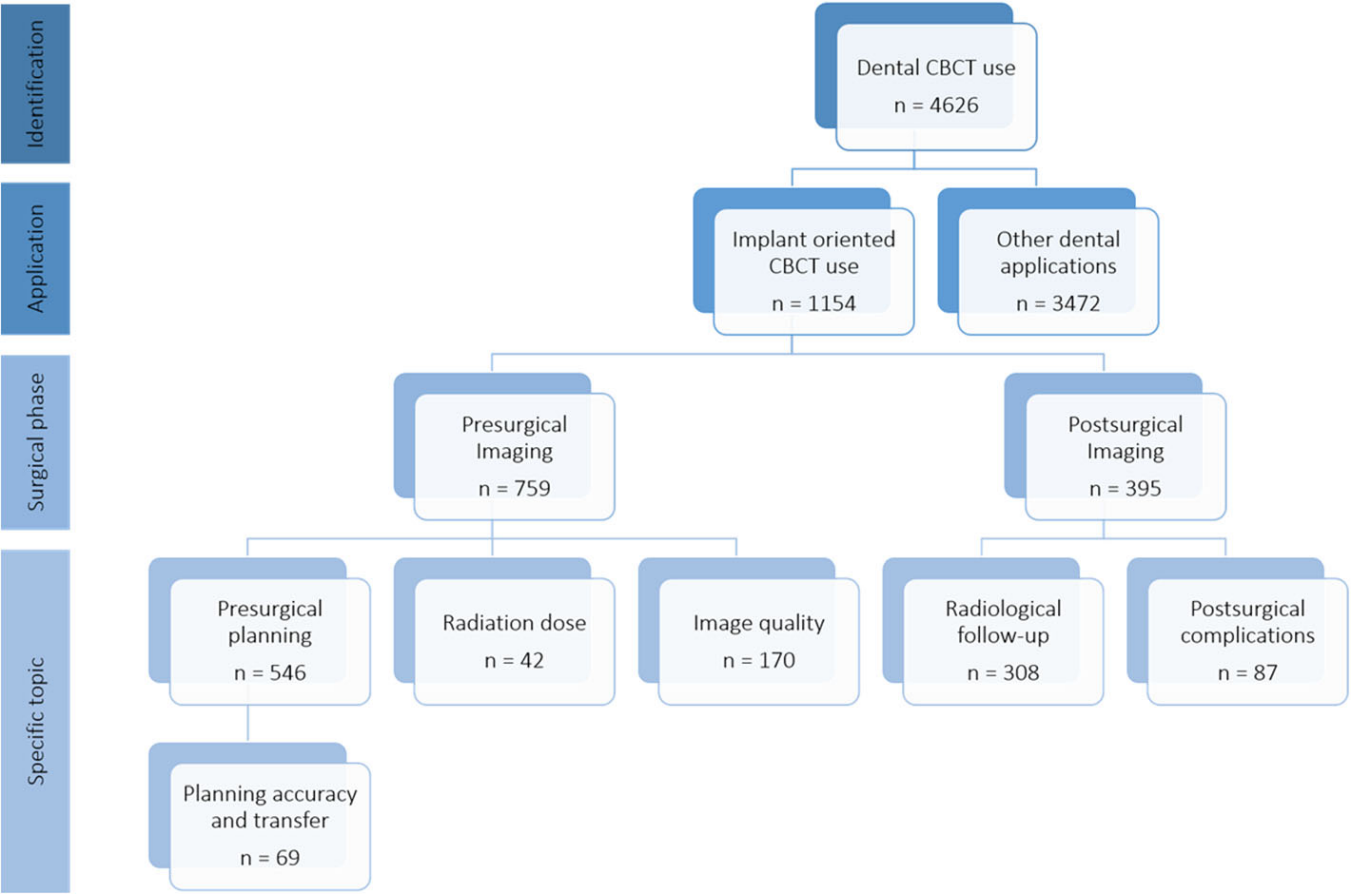
Availability of peer-reviewed articles on the use of CBCT is dentistry and more specifically in the pre- and postsurgical phases of implant dentistry (PubMed output up till November 30 2016). Roughly, every fourth article published on CBCT is related to the use of CBCT in implant dentistry, with two out of three on the presurgical use of CBCT, with a vast majority on the application of CBCT for presurgical planning and transfer to implant placement

**Fig. 2:**
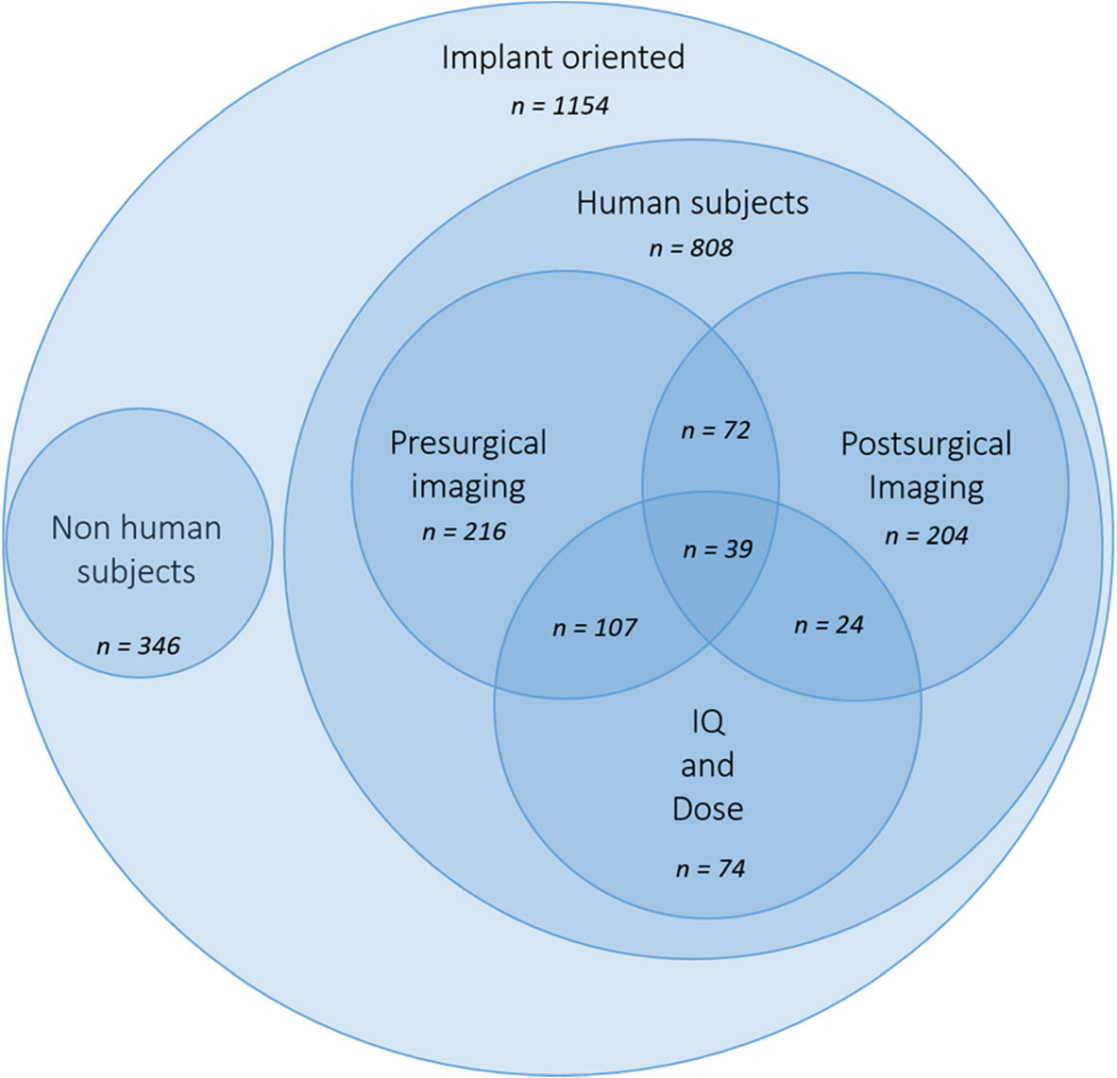
Availability of peer-reviewed articles on the use of CBCT dentistry in implant dentistry, focused on studies performed on human subjects (PubMed output up till November 30 2016). The articles are then divided in three main areas of application: Presurgical and postsurgical imaging and image quality (IQ) and dose evaluation. A Venn diagram was used to highlight the intersections of these research areas

To classify the available literature, specific search queries were used (Table 1). In particular, these queries were combined in order to divide the available literature by specific topics. Figures 1 and 2 show the results of the searches and classification processes.

**Table 1:** Search queries combined in order to classify the available peer-reviewed literature, in MEDLINE (PubMed) database, on the use of CBCT in implant dentistry

ID	Related topic	Search query
#1	CBCT use	cbct OR cone beam computed tomography OR cone beam computer tomography
#2	Implant oriented application	jaw OR teeth OR dental OR dento*
#3	Presurgical imaging	planning or presurgical OR preoperative OR planning or drill guide OR drilling guide OR template
#4	Postsurgical imaging	radiological follow-up OR follow-up or postsurgical* OR postoperative* OR post-operative* OR after surgery
#5	Image quality	image quality OR artifact* OR noise OR accuracy
#6	Dose evaluation	dose OR radiation dos* OR dosi*
#7	Implant planning	planning OR (planning AND (accuracy or accurate or validate or validation or evaluation))
#8	Postsurgical complication	complica* OR nerve OR iatrogenic OR damage OR neuro* OR vascular OR neural

The performed electronic search was complemented by hand-searching, and the final selection of publications was performed after consultation of the working group, consisting of all coauthors of this paper. Disagreements regarding study inclusion were discussed by the investigators. The results of the search process were then summarized in 12 focused questions that identify different areas on the use of CBCT in implant dentistry:

1. *Why to use CBCT in implant dentistry?*
2. *What is the radiation dose level of dental CBCT?*
3. *Which parameters influence image quality in CBCT?*
4. *When to use CBCT in implant dentistry: existing guidelines?*
5. *How to apply CBCT guidelines for the individual patient?*
6. *How to optimize scanning during presurgical use of CBCT?*
7. *How to use dental CBCT beyond radiodiagnostics?*
8. *What are the requirements for creating a virtual patient?*
9. *What are the requirements for 3D model making?*
10. *What should we know about metal artefacts in CBCT?*
11. *How to export and transfer image data?*
12. *When to use CBCT postsurgically?*

**Fig. 3:**
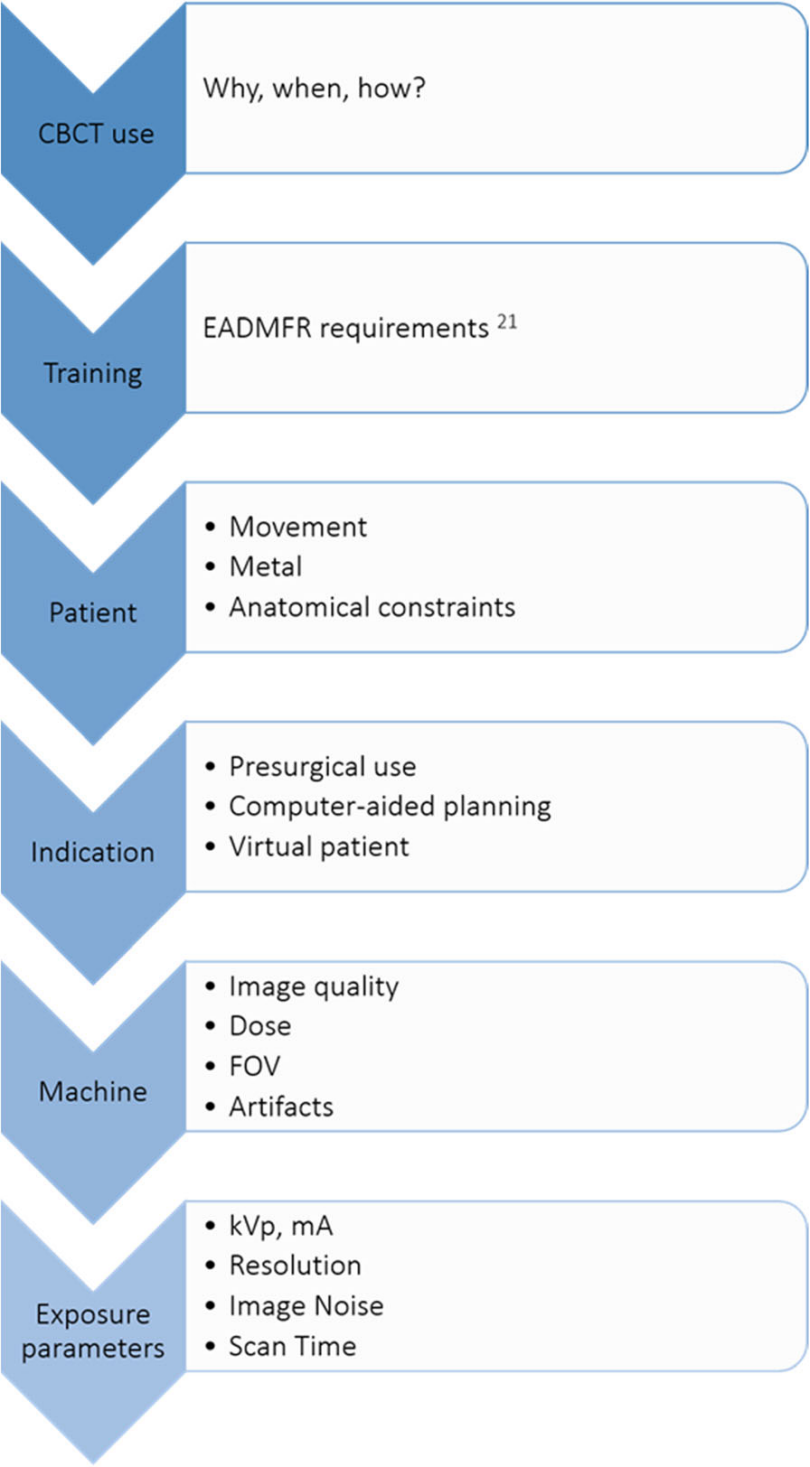
Flowchart of the decision path that clinicians need to follow to find the optimal acquisition set-up of CBCT images in daily practice

These questions trace step by step the decision path that clinicians face in daily practice, see Fig. 3. All together, they represent a series of recommendations that try to integrate the evidence found in the literature with the needs of the clinician.

## Results & Discussion

### Why to use CBCT in implant dentistry?

The first CBCT device was introduced in the late nineties (NewTom 9000, QR, Verona) with the initial scientific reports dating back from 1998 [6]. The overall advantage of using CBCT in implant dentistry is related to its ability to acquire detailed volumetric image data of the maxillofacial region for diagnostic and presurgical planning purposes. Yet, the accessibility of dental CBCT, due to its compact size, reasonable dose, low cost and ease-of-use is probably the prime contributor to its growing success. Since its introduction, the market has been exponentially growing with more than 85 distinct CBCT models readily available. This also includes hybrid or so-called multimodal systems for combined 2D (panoramic and/or cephalometric) and 3D (CBCT) imaging apart from less expensive and primary panoramic machines with a small detector size for scanning narrow field-of-views with a 3D button. CBCT machines are used for diagnostic indications, yet also for presurgical planning and transfer to implant surgery and rehabilitation [1, 5]. The growing interest in dental CBCT use went hand in hand with the growing market for third party software for 3D surgical planning and guidance [2–4]. This is evidenced in Fig. 6, where it becomes obvious that since the introduction of CBCT, there has been a significant increase in publishing scientific papers in relation to dental applications, with roughly a fourth of these studies on CBCT in implant dentistry, following the same upward tendency. Unfortunately, and disregarding the positive trend in publications, a direct consequence of this CBCT revolution and the exponential rise in equipment remains the creation of a clinically significant gap between the existing scientific literature and available hardware and software [7]. It should therefore be stated that research findings cannot simply be generalised as published evidence may often refer to one CBCT machine and not necessarily apply to other equipment [5]. Despite the dedicated properties of CBCT for dentomaxillofacial examinations and its growing use over the last decade, more specifically in implant dentistry, one should realise that there is a enormous variation in radiation doses and image quality and attributed to machine- and protocol-dependent variables [1, 5, 8].

### What is the radiation dose level of dental CBCT?

At this level, it is essential to recognise the close relationship between image quality and radiation dose. It would be simple and straightforward to reduce radiation doses to extremely low levels, but one has to properly investigate that prior to doing so. Indeed, such extreme low dose levels may render images diagnostically useless. In fact, we require diagnostically adequate images for a specific indication. This has evolved in adapting the traditional ALARA principle toward ALADAIP (As Low as Diagnostically Acceptable being Indication-oriented and Patient-specific), as position statement of the Dimitra Research Group [9].

**Fig. 4:**
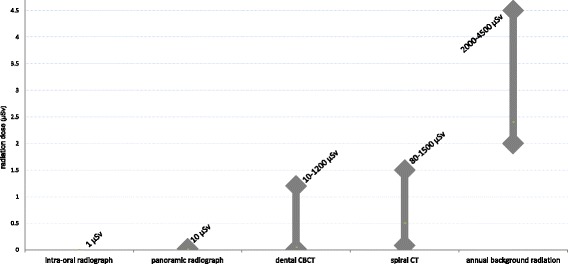
Variation in radiation doses of dental CBCT in relation to dose ranges of other orofacial imaging modalities and natural background radiation

Effective radiation doses for CBCT should be typically far below the levels of spiral CT, thus being considered as a true advantage. It should preferably be an equivalent of 2 to maximally 10 panoramic radiographs (20–100 μSv) [1, 5, 8]. Unfortunately, commercially available CBCT systems seem to vary enormously. Radiation dose levels differ according to the CBCT device being assessed, from around 10 μSv to 1000 μSv (which is an equivalent of 2–200 panoramic radiographs) (Fig. 4). It is noteworthy that even one CBCT may present with a huge range in parameter settings, likewise creating an enormous variation in dose and image quality output [1, 5, 8].

Low dose protocols have been recommended to assist practitioners in optimisation [7, 8]. This has been picked up by manufacturers of CBCT equipment, who introduced low-dose protocols that might even get into the dose ranges of panoramic images. Nevertheless, there is still a need to design studies defining the required image quality in relation to implant dentistry, meanwhile fully balancing the radiation dose output of such image quality requirements [9]. Furthermore, medical imaging is constantly on the move, and thus it should be realised that the dose advantage frequently cited for CBCT compared with mulitslice CT is relative. Depending on the CT generation and the applied exposure protocol, radiation levels for multislice CTs may even be lower than for CBCT scans [8, 10]. This progress in dose optimisation for 2D and 3D technologies demonstrates clearly that radiation dose and related risks are dynamic entities, that need to be frequently monitored and reconsidered.

**Fig. 5:**
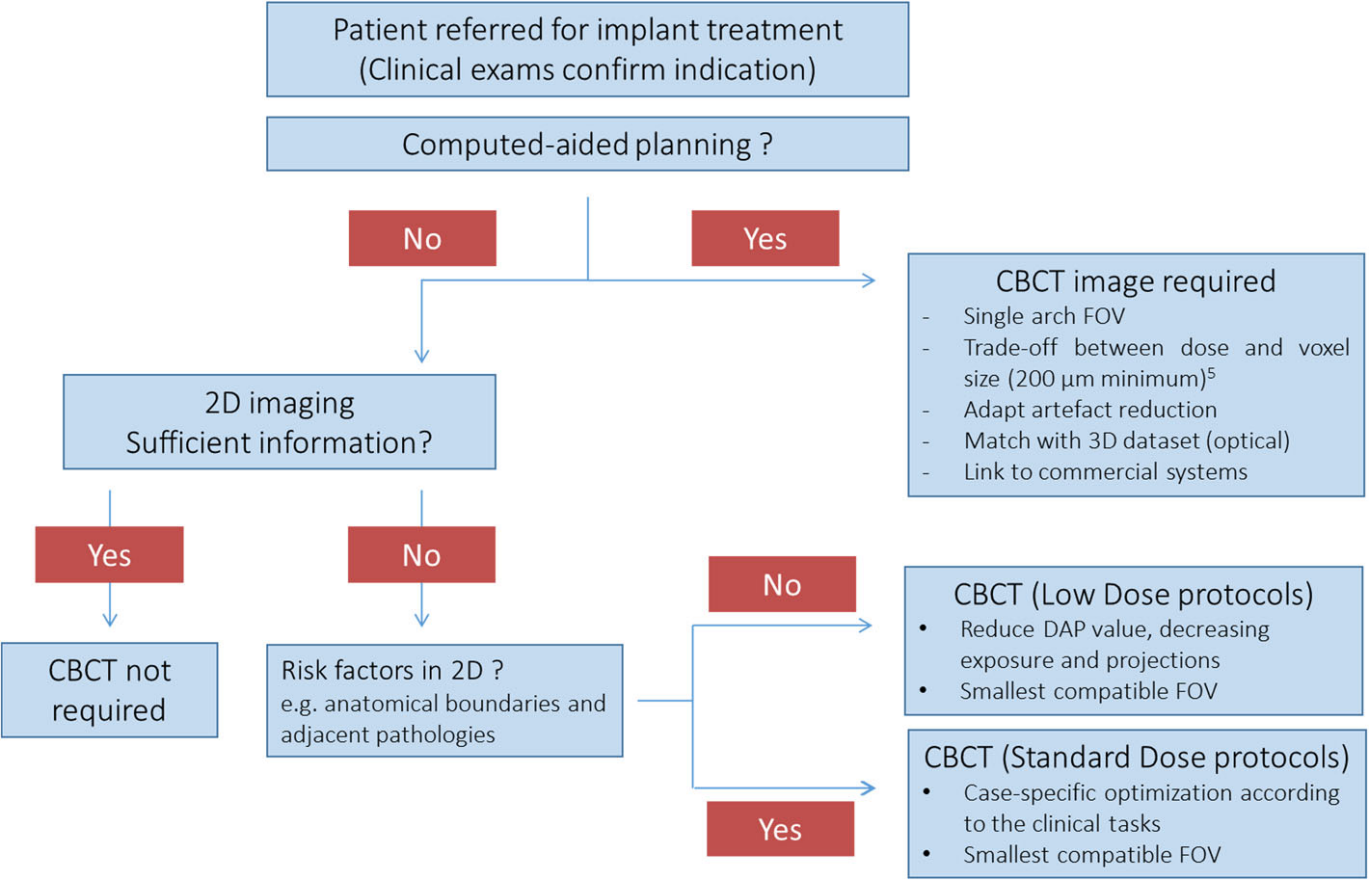
Dose optimization strategy algorithm/flowchart (adapted from [17])

Furthermore, radiation dose levels should be regarded as indication-oriented and patient-specific. Only when respecting the strategy of time-dependent monitoring of indication-oriented and patient-specific radiation doses, a dental practitioner may really comply to ALADIP principles for optimisation and radiation protection in daily practice (Fig. 5) [9].

### Which parameters influence image quality in CBCT?

Image quality performance of CBCT devices may vary widely, similar to, but not only related to exposure protocols and radiation dose ranges [1, 7, 8]. CBCT images are usually considered offering a high spatial resolution with voxel sizes of reconstructed CBCT datasets ranging between 0.08 and 0.4 mm [1]. Small voxel sizes could be diagnostically useful for cases in which small structures such as root canals and periodontal tissues need to be depicted. Variation is also observed when it comes to segmentation accuracy. The latter is a crucial factor when going for an integrated virtual planning including jaw bone models, fabrication of radiographic and surgical guides as well as further prosthetic models. Depending on the CBCT and the parameter settings, a 200 μm accuracy level should be feasible [1, 5]. However, larger inaccuracies may apply (up to 1000 μm and above) [1, 5]. Multi-slice CT often has a better contrast resolution, aiding segmentation and bringing error rates down as compared to CBCT.

Another shortcoming of CBCT is the lack of diagnostically distinct soft tissue contrast, narrowing down the diagnostic potential and hampering applications for soft tissue integration in the presurgical planning. Furthermore, Hounsfield units do not apply to CBCT images, yielding it impossible to compare grey values among or within patients over time [11]. This lack of standardized grey value distribution is complicating the use of CBCT for clinical bone density assessment and follow-up of bone density changes. Hounsfield units (HU) have been designed for medical CT, but do not apply for CBCT [11]. Compared to HU units for medical CT, the reliability of CBCT-based jaw bone density assessment has been found unreliable over time and with significant variations influenced by CBCT devices, imaging parameters and positioning [11]. This lack of HU standardization is a major problem for most CBCT devices. Hitherto, one may question the relevance of this problem when it comes to actual implant dentistry, considering that nowadays a healthy vascularized bone may be more beneficial for implant placement than a sclerotic dense and poorly vascularized bone. What one might thus need instead is a structural bone analysis, like that available in dedicated μCT software. Such structural analysis has already been validated to be used for CBCT imaging, and thus might even have clinical potential for presurgical assessment of bone quality [12]. Also, CBCT images are generally hampered by varying degrees of artefacts expression, mostly deriving from patient motion and from dense restorative materials or a combination of both [1, 8, 13–15].

### When to use CBCT in implant dentistry: existing guidelines?

Guidelines are either consensus-based or derived from a limited methodological approach [8]. One recent systematic review on CBCT guidelines for use in implant dentistry presents an overview of all published guidelines including indications and limitations of CBCT use in implant dentistry [8]. Crucial and still valid are the set of 20 basic principles published in 2009 by the European Academy of Dental and Maxillofacial Radiology (EADMFR) [16]. The set of principles was formulated to act as core standards, useful for reference and adoption to national procedures within European countries and elsewhere. While statements one to eight relate principally to CBCT justification, statements nine to fifteen broadly deal with dose optimisation in relation to image requirements. The last four statements discuss the need for adequate training and competence levels for CBCT use. For diagnostic viewing, a distinction is made between diagnosis in the area of teeth and jaw bones and larger or other anatomical areas. Teeth and jaws may be diagnosed by general practitioners who received adequate training, while specialist training is required for evaluation of larger or other anatomical areas [7, 16].

A more recent publication [17] is based on the sedentexCT guidelines [7] with a further elaboration on the principles for justification and optimization strategies for CBCT use, when dealing with oral implant placement. This document represents the current EAO guidelines for the use of diagnostic imaging in implant dentistry based on a consensus workshop organized by the European Association for Osseointegration in 2011 [17], and contains a revision of the initial EAO guidelines from 2002 [18]. Likewise, a position paper with specific reference to oral implant placement and the potential use of CBCT prepared by the American Academy of Oral and Maxillofacial Radiology was published around the same time [19], being also a revision of the 2000 AAOMR guidelines [20]. Once more, the main reason for both revisions is to be found in the growing role that CBCT started to play over the last decade, particularly in implant dentistry. The main difference in these guidelines is probably the fact that EAO is stressing the needs for adequate and specialist training in relation to the use of CBCT in dental practice, even if simply referring the patient for CBCT. This issue was already eleborated in an additional two documents, namely the sedentexCT guidelines [7] and the EADMFR position paper on training requirements for CBCT use [21].

### How to apply CBCT guidelines for the individual patient?

**Fig. 6:**
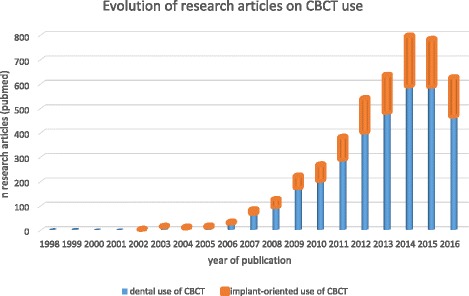
Exponential growth in publishing scientific papers in relation to dental applications, since its first appearance in 1998, with a fourth of the studies on CBCT in implant dentistry, following the same growth tendency (pubmed output up to 2016)

**Fig. 7:**
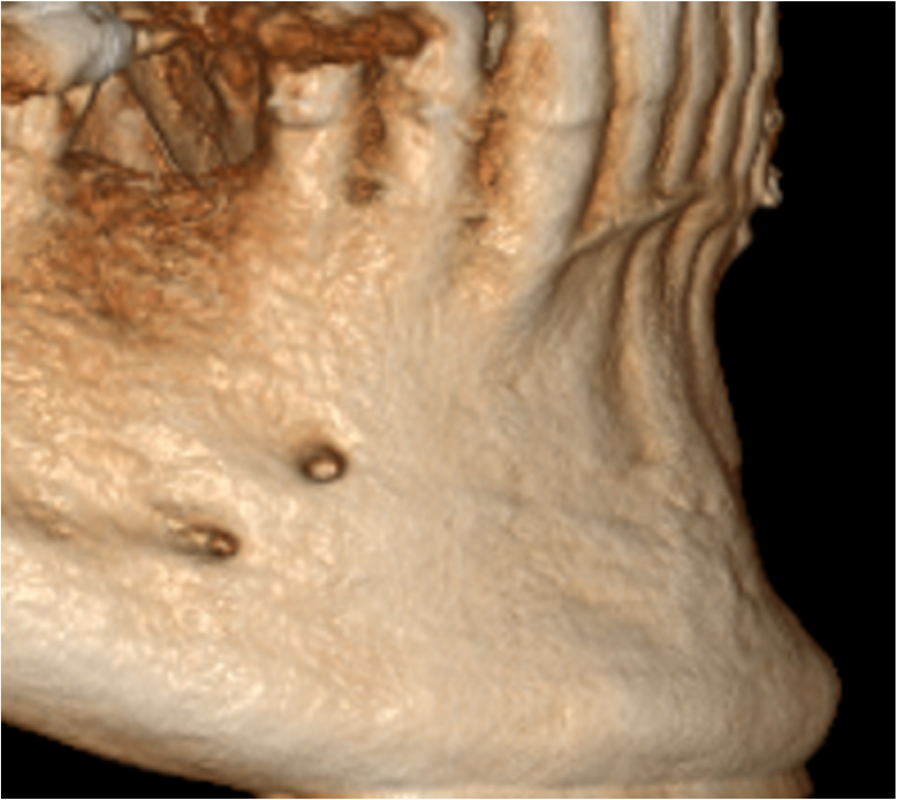
Double mental foramen visible via volumetric imaging of the jaw bone, presenting a risk for nerve damage, if left undetected

In general, when benefits outweigh the risks, CBCT is justified [8, 17]. Since the appearance of dental CBCT, there has been an exponential growth in scientific publications in relation to dental applications, with a fourth of the studies on CBCT in implant dentistry, following the same growth tendency (see Fig. 6). This trend and proportional use of CBCT in implant-related research is matching well with the clinical indications for CBCT use in private practice. Roughly, every fourth article published on CBCT is related to the use of CBCT in implant dentistry, with two out of three on the presurgical use of CBCT, primarily for presurgical planning and transfer to implant placement (Fig. 1). The justification for CBCT use during the preoperative planning phase is based on the need for specific anatomic considerations (identification of anatomic boundaries and morphology, proximity of vital anatomic structures; Fig. 7), esthetic challenges in the anterior maxilla, insufficient bone volume, shape and quality, the use of more advanced surgical techniques (grafting, distraction, zygomatic implants) and the integrated presurgical planning and virtual patient approach (Table 2) [2, 4, 5, 8, 17, 19, 22, 23].

**Table 2:** Presurgical use of CBCT: indications described in guidelines and other scientific reports

Indications for presurgical use of CBCT in literature
Identification of critical anatomic boundaries [8, 17, 19]
Prevention of neurovascular trauma [8, 17, 19]
Specific challenges for the anterior esthetic zones [8, 17, 19]
Borderline cases related to inadequate bone morphology, volume and quality [8, 17, 19]
Augmentation procedures [8, 17, 19]
Special techniques (grafting, distraction, zygoma implants) [8, 17, 19, 22]
Suspected trauma history of jaws and teeth [8, 17, 19]
Doubtful prognosis of neighboring teeth [5, 8, 17]
Presurgical planning and transfer [2, 4, 5, 8, 17, 19, 22, 23]
The virtual patient [23]

One of the most frequent applications of presurgical use of CBCT is the computer-aided planning and transfer for oral implant placement. This approach inevitably requires 3D datasets for further planning in a dedicated software and potential transfer to the surgical field [2, 4, 5, 8, 17, 19, 22, 23]. The advantage of such a digital planning is expected to be an integrated approach of biomechanical, functional, and esthetic aspects to strive for a more predictable outcome, meanwhile avoiding complications [2–4]. Most often, such planning and transfer is realised based on surgical planning and related static drill guides. Fewer surgeons use a dynamic transfer of the planning via a navigaton system. Whilst the latter may be considered somewhat more time-efficient during the presurgical phase, with an additional advantage of real-time visualisation and thus some potential added intra-operative degrees of freedom, its widespread use in clinical practice might be self-limiting. This is mainly related to the complex set-up during surgery, the need for an additional and sophisticated peroperative tracking system, requiring calibration of the surgical field and recalibration upon jaw motion, while being restricted by the mouth opening (surely for posterior implants), with additional bounds to accessibility of the surgical field caused by the added appliances of the tracking system [3]. A meta-analysis on the accuracy of computer-aided implant planning and transfer revealed implant placement with a mean error of 1 mm (up till 6.5 mm) at entry and 1.2 mm (up till 7 mm) apically with an average angular deviation of 3.8° (up to 25°). Less deviation was found when using static surgical guidance, preferrably with a single surgical template and more fixation pins. Overall, computer-guided implant placement can be considered accurate, if carefully performed. Errors during CBCT imaging, planning and surgical transfer may however lead to significant and clinically unacceptable deviations [3].

Postoperative use of CBCT in implant dentistry represents a minority of the applications (see Fig. 1) and will be discussed later.

### How to optimize scanning during presurgical use of CBCT?

CBCT images are composed of isotropic and high resolution voxels (from 0.08 to 0.4 mm depending on device and acquisition parameters), providing the essential prerequisites for image resolution and high signal to noise ratio for presurgical planning of implant placement [5]. However, small voxel sizes may cause a reduction in contrast-to-noise ratio, meanwhile requiring higher radiation exposure levels to the patient [1, 13]. This implies a need for optimization, by balancing patient-specific requirements with indication-oriented settings and machine-dependent parameters (ALADA). Yet, the lack of standardization among CBCT systems leads to a wide difference in acquisition parameters and performances among different devices, making it extremely difficult to generalize research finding and standardize imaging requirements for computer aided surgical planning [1].

Considering that there may still be room for optimization (See Fig. 5), this paragraph lists some clinical tips and tricks optimizing CBCT scanning for presurgical planning rendering its transfer to surgery more predictable. CBCT imaging should always be carried out while maintaining the correct balance between cost and radiation dose on one hand and required clinical information on the other hand. Protocols should be patient-specific and indication-oriented [1, 5, 8, 9, 16, 17]. This strategy is heavily influencing the radiation dose output as no machine is producing one standard dose for all patients and all indications. Depending on patient age and anatomy as well as the requirements for the specific diagnostic tasks, the field of view should be individualized, the required resolution adapted and the patient conditions carefully observed.

A stressed or easily moving patient might be advised closing the eyes to avoid motion artefacts. The presence of restorations, implants, metal posts and endodontic obturations may significantly hamper the image quality and thus the segmentation procedure during model making for presurgical planning and transfer. The negative impact of artefacts on segmentation may be reduced by adjusting the field of view during scout viewing prior to actual CBCT scanning to avoid image acquisition of metal-containing diagnostically unuseful areas (e.g. by deselecting the clinical crown level; by removing a prosthetic structure not rigidly fixed in the mouth; by strictly limiting the field of view area to the jaw bone involved). Further reference to the factors causing metal artefacts and their clinical applications are listed below in a separate paragraph.

For surgical guide fabrication, scanning at 200 μm resolution may be sufficient [1, 5]. Selecting a higher resolution scan may lead to more noise and sometimes even more scattering hampering the segmentation process of the anatomic jaw bone and the registration of a 3D impression scan on top of the 3D jaw model. During CBCT scanning with a radiographic scanning template, it is required to check for perfect fit at the occlusal level or on the soft tissues of edentulous jaws. Scanning templates should not be fabricated from a high-density material and/or not be too thick. The same applies for the occlusal splints which may be very useful separating upper and lower jaw models when there is a need for distinct jaw bone segmentation. Cotton wools are easy-to-use solutions for keeping lips and cheeks away from the gingiva when there is a need for assessing gingival thickness at the vestibular bone site [24].

Presurgical planning and transfer is often a multistep approach, with each step contributing to the resulting discrepancy between the original presurgical planning and the final outcome result following implant placement. When opting for such a procedure, it is necessary to take into consideration the proper time for optimised CBCT image acquisition, diagnostic interpretation, data manipulation, volume segmentation, registration or fusion of distinct 3D datasets, integrated planning and further accurate transfer to enable a predictable outcome, meanwhile avoiding complications. There is an urgent call for simplification of this complex and lengthy procedure, involving less steps as this may lower the threshold for clinicians to opt for such a digital approach, meanwhile decreasing the number of potential errors inherent to each of these individual steps [25].

### How to use dental CBCT beyond radiodiagnostics?

Apart from its radiodiagnostic potential, dental CBCT may present a further treatment potential, such as the potential for surgical guides and prosthetic rehabilitation via CAD/CAM procedures [23, 26–31]. The large variations within CBCT units may lead to variable degrees of linear, diagnostic and 3D model accuracy, which are all needed to refine diagnostic tasks, surgical planning and CAD/CAM transfer. Nowadays, studies are focusing on overcoming technological shortcomings by assessing optimisation of exposure protocols or by registration of CBCT scans with optical datasets to eliminate the drawback of CBCT-derived metal artefacts [26, 30]. These optical datasets are derived from 3D optical cameras, which may offer the possibility to avoid traditional impression-taking. This may help to eliminate the necessity of intra-oral impression materials and subsequent fabrication of dental casts, meanwhile reducing time and handling errors inherent to these procedures. The available intraoral 3D scanners may offer excellent accuracy (up to 10 times better than CBCT), while being more comfortable for the patient than conventional impression-taking and far more efficient for the daily workflow [23, 26, 30]. By image-fusion with basic CBCT data, a digital cast with an accurate surface would thus enable further treatment using integrated 3D dataset for patient-specific CAD/CAM procedures [23, 30, 31].

This semi-automation may eliminate manual steps and inevitable human errors when producing dental restorations [32–34]. Continuous developments may evolve to further simplification and automation, with less chair-side time and fewer recall visits for the patient at an expense of more computer time for the practitioner. This may then result in virtual model creation to allow for patient-specific customization of oral implant rehabilitation [29].

Meanwhile, it should also be mentioned that integrated 3D facial scanning has become available for various CBCT units. The latter implies a concomitant 3D laser acquisition of facial soft tissues during CBCT scanning. This may then allow for a fully integrated planning with the 3D facial tissue scan registered onto the bony skull image, which may contribute to enhancing planning efficiency and prediction of the treatment outcome [28, 32, 35]. It may also create opportunities for surgical case follow-up, free of ionising radiation.

### What are the requirements for creating a virtual patient?

The aforementioned evolution towards the concept of a virtual patient for dental implant surgery, creates new challenges for appropriate CBCT scanning. The virtual patient is a digital record that is used to plan the ideal implant position with respect to aesthetic, prosthetic and surgical requirements. It integrates information (datasets) obtained from facial scanning technology, digital intra-oral impressions and CBCT imaging in one virtual coordinate system [23, 28, 31, 32]. The virtual teeth arrangement is planned with regards to aesthetic and prosthetic functional requirements so that the ideal prospective implant position can be identified [29, 33]. Subsequently, a surgical guide is fabricated for fully guided implant insertion, and the treatment can then proceed with creating the provisional and final prosthesis using the same pre-operatively planned prosthetic setup [29, 30, 32, 33]. There are several imaging requirements to achieve this functional prosthetically oriented surgical goal, which are related to the accuracy of each scanning modality as well as to the fidelity of the image integration procedure.

Three-dimensional (3D) facial scanning provides information regarding the external soft tissue profile in three dimensions, and it can be of tremendous aid during the 3D digital smile design phase of the treatment [28]. In prosthodontics, facial scanning needs to provide high resolution 3D mesh data and photorealistic texture rendering in order to permit a virtual clinical evaluation phase. Several facial scans in neutral head position, maximum smile and using cheek retractors need to be obtained and merged together and the labial surfaces of the anterior teeth should be clearly depicted to allow registration with the digital dental model scans [28, 33, 35, 36]. In order to integrate facial scanning with CBCT, the forehead region needs to be clearly visible in both scans. This approach has been previously validated and the accuracy was found within clinically acceptable limits [35]. Recent studies demonstrated the applicability of the virtual patient approach to preoperatively plan the surgical placement of dental implants and to CAD-CAM design and fabricate screw retained prosthesis in partially and completely edentulous patients [31, 35, 36].

Several factors influence the accuracy of guided surgery systems related to CBCT image accuracy such as type of tissue support, flap approach, free or full guided implant insertion [36–40]. In partially edentulous cases, the surgical guide is fitted onto the teeth (tooth-supported). The imaging requirements of CBCT in these cases include high fidelity depiction of the dental arches including both teeth and alveolar process. However, CBCT limited spatial and contrast resolution impede accurate 3D reconstruction of the teeth to allow for the fabrication of a surgical guide [3, 27]. Therefore, CBCT 3D models are integrated with digital teeth models obtained using digital intraoral impressions at a spatial resolution of around 50 μm [39]. Recent research in this area demonstrated that this approach is valid and can be applied in the daily routine although the accuracy of CBCT 3D models of the teeth remains largely influenced by scan field of view and resolution in addition to the presence of artefacts originating from metal or zirconia structures [41]. The presence of pre-existing metal or zirconia prosthetic work largely deteriorates the visibility of the teeth on CBCT scans, thereby impeding accurate integration (registration) with teeth models obtained from digital intraoral impression. A significant negative influence of metal artefacts on the 3D teeth visibility in CBCT, and therefore on the overall accuracy of guided surgery planning was recently reported [40, 42]. As has been previously suggested in orthognathic surgery literature, a workaround to solve this imaging requirement is to artificially install landmarks in a radiographic template or directly on the gingiva to act as a common fiducial reference for the registration procedure [43]. Currently, radiographic templates could also be CAD designed using the information obtained from intraoral scanning and can be 3D milled or printed thereby eliminating the need for a conventional impression [44].

In complete edentulism, the situation is drastically different due to the absence of teeth as a fixed common reference. Typically, a radiographic template containing the ideal prosthetic setup would be CBCT scanned together with the patient and the resulting surgical guide would be either bone or gingiva supported. Beside the general requirements for CBCT image quality to visualize vital anatomical structures, the scan data should also be able to provide good visibility for the radiopaque fiducial markers in the radiographic template and preferably, the outer contour of the gingiva should also be made visible to facilitate accurate implant planning [45]. Perhaps the most promising development in recent years is the mini-implants supported surgical guide placement in completely edentulous patients. Using this approach, the guide is screw retained on mini-implants thereby eliminating the inaccuracies incurred from bony and gingival support [46]. In addition, the mini-implants can be used to fabricate a radiographic template, which can also be employed for creating a virtual teeth try-in. However, studies on this approach are still lacking.

Another important element in the surgical implant virtual patient approach is the virtual articulator, which is used to simulate the dynamics of the patient’s jaw movement during chewing, swallowing, breathing, bruxing and speech. Several techniques are currently available to virtually mount the upper cast in the correct coordinate system with reference to the skull base using the kinematic or hinge axis of the temporomandibular joint (TMJ) as a neutral zero position [47, 48]. An important requirement here is the Euclidean dimensional correspondence between the starting point of the jaw motion tracing device and the virtual articulator. More development on this front is necessary. Towards the future, reconstructions from MRI might potentially get integrated in the virtual patient model, which could be particularly interesting for dynamic TMJ evaluation.

### What are the requirements for 3D model making?

The accuracy of templates strictly depends on virtual 3D model accuracy. Poor image resolution may result in insufficient image quality. The latter is the main cause of 3D model inaccuracy, since it amplifies the effects of all image processing approximations [49].

As for teeth, occlusal surfaces require a five to tenfold better image resolution than the lowest voxel size levels of CBCT. On top of that, tooth restorations may increase the inaccuracy level by artefact formation. To overcome this limit, recent studies try to compensate the lack of information on teeth morphology by fusing the CBCT surface model with a digitalized dental model acquired with optical systems [26–28, 30].

Surface accuracy is also related to the Field of View (FOV) used during the acquisition step. Different studies concluded that images acquired with medium and small FOV allow to obtain more accurate models compared to those acquired with a large FOV [50, 51]. Nevertheless, small FOVs show more pronounced artefacts and wider grey level variability compared to larger FOV scans [50]. Model accuracy is also depending on the segmentation process. Currently, in dentomaxillofacial applications, segmentation is mainly performed by expert operators using manual thresholding [50, 52]. This approach is very subjective and strictly related to operator experience. In addition, anatomical structural variations in the craniofacial region require to develop specific segmentation approaches [50, 53, 54]. To reduce operator dependency and improve segmentation accuracy, some fully or semi-automatic segmentation approaches were developed [52, 55].

In conclusion, image segmentation, can deeply affect 3D model accuracy, even if generated from high-resolution image data. For this reason, surgeons that use virtual planning must have a deep knowledge of the imaging techniques involved in the presurgical work-up [49]. Only adequate training will allow them to set the optimal acquisition parameters and post-processing steps to improve model accuracy and consequently patient treatment.

### What should we know about metal artefacts in CBCT?

CBCT images are often corrupted by artefacts, which are defined as visualized structures in the reconstructed data that are not present in the scanned object [41]. In particular, the presence of dense materials, such as metals, causes different kind of artefacts in CBCT images. Among these the most common are: beam hardening, extinction and exponential edge gradient effect artieacts [56].

The presence of these artefacts affects image quality in several ways, ranging from bright streaks radiating from the metallic object, dark areas near it to the complete loss of information between adjacent dense objects [13, 14]. This group of artefacts represents the so called “metal artefacts”.

The presence of such artefacts in CBCT compromises diagnosis and surgical planning. Material density and exposure parameters play a key role in artefact expression. Pauwels et al. quantified the impact of different CBCT devices and exposure protocols on the expression of metal artefact induced by titanium implants, with an advice to develop optimized exposure protocols adequate for metal artefact reduction [14]. Material density, design and composition yield a variable radio-opacity, with a strong effect on the amount of artefacts, due to the inability of the X-ray beam to pass through the imaged object and the consequent insufficient number of photons that reach the detector [41]. Based on the increased material density as compared to titanium, zirconium dioxide implants might thus generate stronger artefacts as compared to other materials.

Due to the clinical relevance of this matter, several efforts were made to reduce metal artefacts in CBCT images. A recent study conducted by Kuusisto et al. demonstrated that composite materials give less artefacts, finding the cut-off point of artefacts at 20% radio-opaque filling material in composite implants [56].

Another approach to reduce metal artefact is to implement specific metal artefact reduction (MAR) algorithms, which allow improving image quality. These correction algorithms can be classified in three different groups: interpolation-based methods, iterative reconstruction approaches and adaptive filtering algorithms [57]. In the last years, specific MAR algorithms for CBCT images were developed [45, 46, 57–61], and now MAR solutions are available in most of the commercial devices. Bechara et al. evaluated the performance of some MAR commercial solutions measuring image quality parameter such as contrast-to-noise ratio and the gray value variation concluding that the tested MAR solution were able to improve image quality [62, 63].

However, only a few studies evaluate the clinical applicability of these artefact reduction methods. The presence of metal artefacts can affect the visualisation of the periimplant bone. This may lead to a biased and/or erroneous diagnosis and therefore also to inappropriate treatment decisions [64].

Unfortunately, as mentioned before, the lack of standardization among CBCT device settings makes it difficult to generalize these findings. Nevertheless, it seems that right now MAR solution do not add diagnostic information, even if image quality parameters are improved.

### How to export and transfer image data?

DICOM (Digital Imaging and Communication in Medicine) was originally developed to create a worldwide norm for digital image acquisition, storage, and display in medicine, and also to have a standardized method for transmission of medical images and related information on patient and technical image parameters. “Digitization” is increasingly widespread in dental medicine in terms of radiographic image acquisition (2D and 3D), optical surface scanning (intra- and extraoral), CAD/CAM systems, and the electronic charting of patient records. Unfortunately, the DICOM standard is not really fully implemented in oral health care, with primarily hospital and dental school settings complying with the standard [65].

Picture archiving and communication systems (PACS) software act to integrate image acquisition, storage, retrieval, and viewing based on the DICOM standard. In dentistry, the use of PACS is often limited to hospital facilities, larger dental clinics and academic centers [66]. Newer standard dental digital imaging devices including intraoral digital radiographic systems, panoramic views, and CBCT scanners in large part are mostly DICOM compliant. Nevertheless, standards for DICOM compliance for some devices including CBCT and CAD/CAM systems and their interoperability with respect to PACS have not yet been fully established. To facilitate standard communication, accurate image data exchange and 3D data integration into a virtual model, radiographic devices and third-party dental implant software applications should be forced to offer fully compliant DICOM data export [8, 67]. The huge data volume of CBCT DICOM images requires high storage capacity. To save storage space, DICOM viewers usually compress DICOM imges into smaller files during file export [68]. This compression is done using specialized algorithms and can be performed using both lossy or lossless methods [69]. Lossy compression permanently eliminates redundant information, but can result in eccessive image degradation [68]. In order to avoid loss of information, it is advised to use the original data or data compressed with lossless compression algorithms when transferring information to a third-part software. Unfortunately, in literature there is a lack of studies validating the process of image conversion from a proprietary format into DICOM format. Further studies are needed to quantify the amount of information lost during DICOM export, in order to make clinicians aware of this source of error, on top of all other possible sources of inaccuracy during computer aided surgical planning. It is clear that for most CBCT systems there is a diagnostic data loss upon DICOM export and potentially even a further loss when imported into a PACS system or third party software. Indeed, most third party softwares have some additional filtering (e.g. smoothening) at the import phase, which may result in additional information loss. It is therefore recommended to do presurgical diagnostics in the dedicated CBCT-software of the imaging device, prior to export for presurgical planning purposes.

Other challenges in the digital data flow include the fact that there is a growing availability of non-DICOM 3D imaging data formats required to be used for an integrated virtual patient dataset [23, 28, 38, 39]. Examples include STL and OBJ formats, respectively, used for digital intraoral impressions and printing as well as for facial scanning. Transferring those datasets to PACS systems is actually not possible, as such that the power of the integrated virtual patient information is lost at this level.

### When to use CBCT postsurgically?

**Fig. 8:**
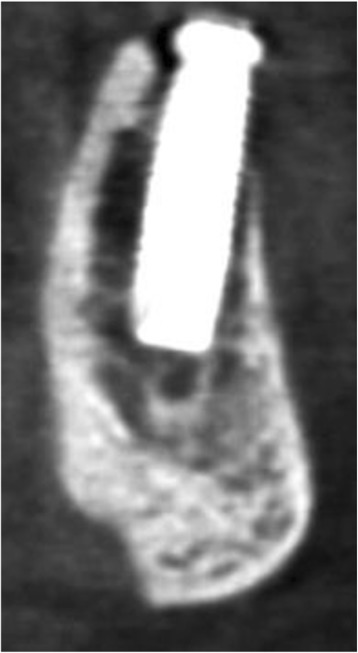
Implant in the mandible showing some beam hardening artefact, and in addition a position at the roof of the incisive canal, causing neurosensory disturbances

Postoperatively, CBCT is used for evaluation of graft healing, to assess complications mostly related to neurovascular trauma and when implant retrieval is anticipated, as shown in Table 3 [5, 8, 17]. More than a fifth of the articles (22%) dealing with postsurgical CBCT scanning are related to postsurgical complications (see Fig. 1). While implant placement accounts for only 3% of cases with neurosensory disturbances, when focusing on cases with a permanent neurosensory deficit, implant placement is responsible for a four-fold number of cases (12% of all iatrogenic permanent trigeminal injuries) [70] (see Fig. 8). Besides, implant surgery carries a risk to cause severe intraoral hemorrhage. For mandibular implant placement specifically, multiple reports have been documenting life-threatening upper airway obstruction after postoperative bleeding in the mouth floor [71]. According to a recent study of Yilmaz et al. [72], an inadequate radiological assessment is the most common reason for postsurgical neural injury. This finding supports the need for proper training on CBCT use and its diagnostic interpretation, even when being a simple referrer for CBCT imaging [21].

**Table 3:** Postsurgical use of CBCT: indications described in guidelines and other scientific reports

Indications for postsurgical use of CBCT in literature	Needed 3D info	Drawback CBCT
Postsurgical complications (e.g. neurovascular trauma) [8, 17, 19]	Evaluate location and severity of problem and how to approach	Artefact by implant may mask neurovascular bundle CBCT fails to visualize neurovascular bundle
Healing follow-up of complex surgical procedures [8, 17, 19]	Check bone healing and volumetric outcome	Detrimental artefacts of implants in borderline case (pneumatised maxillary sinus with inadequate bone)
Maxillofacial trauma with suspected complications at the implant level [8, 17, 19, 22]	Check mechanical failure implant or superstructure	Diagnostic failure to spot trauma caused by metal artefacts
Retrieval of osseointegrated implants (infectious or mechanical failure etiology) [8, 17, 19]		Blooming of implant masking neurovascular structures

For mere follow-up of implant placement, CBCT seems not be the ideal diagnostic tool. Up till now two-dimensional intra-oral radiographs are still considered the prime tool when there is a clinical need for postsurgical implant monitoring [73]. In contrast to continuously moving concepts and revolutionary changes in implant dentistry, no consensus conference has ever questioned the 2D intra-oral peri-implant bone level measures [8, 17]. Yet, considering that we are nowadays more and more focussed on bone grafting for defect fill up and sinus augmentation, vestibular bone in the esthetic zone, severe peri-implant bone loss (e.g. peri-implantitis), alternative implant and abutment designs, we should question the traditional two-dimensional imaging diagnostics. Realising that we need to evaluate three-dimensional bone healing, including morphological, volumetric and trabecular remodelling, one could wonder what could be observed and diagnosed by merely looking to 2D approximal peri-implant bone. We should admit that marginal bone levels only reflect a few μm of observation along a peri-implant circumference between 6 and 13 mm long. The only way to fully grasp the peri-implant tissues, is indeed to obtain a true 3D view of the clinical situation, which brings us back to three-dimensional imaging of the peri-implant bone [74, 75]. It is hoped that towards the future, CBCT hardware and software may overcome the current limitations and as such to allow for clinically realistic peri-implant diagnostics.

Although the CBCT market has been rapidly growing, few companies seem to have paid enough attention to the huge problem of metal artefacts, surely when it comes to presurgical implant planning and peri-implant follow-up [13, 14, 40]. Artefacts are even worse with denser material and thus more pronounced with implants in zirconium than in titanium [76]. In general, such materials cause blooming of the implant with enlargements easily reaching a third of the implant diameter, apart from other artefacts such as streaks and black bands [76]. In areas where the thin vestibular bone plate needs to be observed, blooming artefacts may bias diagnosis. Only a few CBCTs and specific protocols seem to allow a reasonably improved peri-implant depiction [76], but still with some patient-specificity. Another point of attention with CBCT use is the lack of standardised grey level calibration or hounsfield scoring, making the comparative follow-up of bone healing, grafting and implant placement rather difficult and quite unreliable [11]. Monitoring pathological changes or quantification of bony healing is possible, yet at the moment, not always possible considering artefact expression and often too complicated for widespread use in clinical practice. Thus and as for now, we need to remain with peri-implant 2D bone level measures using strictly paralleling intra-oral radiographs, even when realising that we may only visualise and measure a glimpse of the entire defect.

## Conclusions

CBCT imaging is a well-established radiographic modality in treatment planning for dental implants, becoming increasingly popular and globally used in oral health care. This is partially due to new insights into anatomic landmarks, and structures at risk during implant placement such as neurovascular structures. Another reason for the growing use of CBCT scanning is the increasing popularity of computer-guided surgery that relies on digital planning based on high-quality CBCT images [38], but may also include the superimposition of intraoral scans and extraoral face scans to create a 3D virtual dental patient [36, 37]. The virtual patient concept is actually demonstrating again the need for standardisation of image data formats enabling a smooth integration of all available datasets (DICOM, STL, and OBJ files) into a craniofacial virtual reality model.

The use of CBCT imaging following insertion of dental implants should be restricted to specific post-operative complications (such as iatrogenic neurovascular trauma), required implant retrieval and follow-up of complex surgical procedures. While to fully grap the peri-implant tissues, is to obtain a three-dimensional view or the peri-implant tissues. And that brings us back to the clinical and the potential means for three-dimensional evaluation For long-term maintenance and follow-up of dental implants, we are still forced to remain with peri-implant bi-dimensional bone level measures on correctly taken periapical radiographs, even if had has no true prognostic value and considering that only the proverbial tip of the iceberg of the actual size and morphology of a defect seen.

The variation in CBCT performances related to radiation doses and image quality, emphasizes the need for more research to establish proper solutions for three-dimensional imaging following the ALADA principle, whilst focussing on artefact reduction caused by motion and metal. A further standardisation is needed for the grey level output as such to be able to assess bone healing, follow-up and evolution of pathological processes. Finally, lossless standard image communication as well as smooth and accurate integration of multiple image datasets, beyond the borders of CBCT, is another point of attention for future developments towards digital dentistry and the creation of an integrated virtual patient.
